# The effectiveness of text messages support for diabetes self-management: protocol of the TEXT4DSM study in the democratic Republic of Congo, Cambodia and the Philippines

**DOI:** 10.1186/1471-2458-13-423

**Published:** 2013-05-01

**Authors:** Josefien van Olmen, Grace Marie Ku, Maurits van Pelt, Jean Clovis Kalobu, Heang Hen, Christian Darras, Kristien Van Acker, Balthazar Villaraza, Francois Schellevis, Guy Kegels

**Affiliations:** 1 Department of Public Health, Institute of Tropical Medicine, Brussels, Antwerp, Belgium; 2 Department of General Practice & Elderly Care Medicine, EMGO Institute for Health and Care Research, VU University Medical Center, Amsterdam, The netherlands; 3 Veterans Memorial Medical Center, Quezon, Philippines; 4 MoPoTsyo, Phnom Penh, Cambodia; 5 Memisa, Kinshasa, DR Congo; 6 Memisa, Brussels, Belgium; 7 Diabetologist, working at Algemeen ziekenhuis Heilige Familie, Reet & Centre de Santé des Fagnes, Chimay, Belgium; 8 NIVEL (Netherlands Institute for Health Services Research), Utrecht, Netherlands & Department of General Practice and Elderly Care Medicine/EMGO Institute for Health and Care Research VU University Medical Center, Amsterdam, The Netherlands

## Abstract

**Background:**

People with diabetes find it difficult to sustain adequate self-management behaviour. Self-Management Support strategies, including the use of mobile technology, have shown potential benefit. This study evaluates the effectiveness of a mobile phone support intervention on top of an existing strategy in three countries, DR Congo, Cambodia and the Philippines to improve health outcomes, access to care and enablement of people with diabetes, with 480 people with diabetes in each country who are randomised to either standard support or to the intervention.

**Design/methods:**

The study consists of three sub-studies with a similar design in three countries to be independently implemented and analysed. The design is a two-arm Randomised Controlled Trial, in which a total of 480 adults with diabetes participating in an existing DSME programme will be randomly allocated to either usual care in the existing programme or to usual care plus a mobile phone self-management support intervention. Participants in both arms complete assessments at baseline, one year and two years after inclusion.

Glycosylated haemoglobin blood pressure, height, weight, waist circumference will be measured. Individual interviews will be conducted to determine the patients’ assessment of chronic illness care, degree of self-enablement, and access to care before implementation of the intervention, at intermediate moments and at the end of the study.

Analyses of quantitative data including assessment of differences in changes in outcomes between the intervention and usual care group will be done. A probability of <0.05 is considered statistically significant. Outcome indicators will be plotted over time. All data are analysed for confounding and interaction in multivariate regression analyses taking potential clustering effects into account.

Differences in outcome measures will be analysed per country and realistic evaluation to assess processes and context factors that influence implementation in order to understand why it works, for whom, under which circumstances. A costing study will be performed.

**Discussion:**

The intervention addresses the problem that the greater part of diabetes management takes place without external support and that many challenges, unforeseen problems and questions occur at moments in between scheduled contacts with the support system, by exploiting communication technology.

**Trial registration:**

ISRCTN86247213

## Background

Diabetes Self-Management Education (DSME) is defined as “the on-going process of facilitating the knowledge, skills, and ability necessary for diabetes self-care”. The overall objectives are “to support informed decision making, self-care behaviours, problem solving and active collaboration within the health care team and to improve clinical outcomes, health status and quality of life” [1]. Literature shows that it is difficult for patients to stay motivated to sustain self-management behaviour and that most patients need on-going support [2]. Owing to the chronicity of the condition, many challenges, unforeseen problems and questions occur at the ‘in-between moments’ outside the contacts with the health care providers [3]. Diabetes Self-Management Support (DSMS) is defined as “activities to assist people with diabetes to implement and sustain the on-going behaviours needed to manage their illness. It includes activities such as education, reminders and behavioural support” [1]. The implementation of DSME and DSMS strategies and its potential positive effects have been described mostly in sophisticated health care settings in high income countries [4-8]. These programmes use a mix of tools to reach patients, such as written information, phone calls and short messaging services and visits. Mobile technology can be particularly beneficial for the management of a chronic disease like diabetes, for instance by supporting behavioural change and reminders for taking medication and for appointments with care providers [9-11]. Overall, the evidence on the feasibility and advantages of the use of mobile technology is positive, but many studies are small and evidence on its effectiveness is not very robust [12-16]. Despite a growing number of studies about mobile phone applications in low income countries [17,18], we are aware of only one publication about a feasibility study assessing the use of mobile phones for diabetes support in such context. This study showed the feasibility of mobile phone use for peer support and health messaging. The participants reported positive effects of the intervention, for instance increased social support coping, yet their physical parameters did not improve in the 6 months’ follow-up [19]. The literature on the use of mobile technology for supporting self-management also points out the need for more process evaluation in order to better understand under which conditions and why such interventions work.

This study will address these gaps, by evaluating the effectiveness of a mobile phone DSMS intervention on top of an existing DSME strategy in DR Congo, Cambodia and the Philippines, using a randomised controlled design with a follow-up of 24 months. The project aims to evaluate not only the effectiveness of the intervention in each country, but also to assess the processes and contextual factors that influence the implementation of mobile phone technology for supporting self-management in order to understand how it works, for whom, under which circumstances.

## Methods

### Objectives

The primary aim of this study is to evaluate the effectiveness of a mobile phone DSMS intervention in addition to an existing DSME strategy in three countries, Democratic Republic of Congo (DRC), Cambodia and the Philippines, to improve health outcomes (HemoglobinA1C (HbA1C) level, blood pressure, Body Mass Index (BMI), waist circumference and diabetic foot problems), access to care (failure-to-attend rate, perceived quality of care and health care expenditure) and enablement (knowledge, self-management and feeling of coping) of people with diabetes participating in a diabetes self- management education programme. The secondary aim is to identify barriers and facilitating factors, including additional cost via an incremental cost effectiveness analysis, for the implementation of mobile phone technology for supporting self-management in low-to-middle income countries.

### Study design

The study consists of three sub-studies with a similar design in three low-to-middle income countries, which will be independently implemented and analysed. The design is a two-arm Randomised Controlled Trial (RCT), in which a total of 480 adults with diabetes (type 2 or 1) participating in an existing DSME programme in each country, will be randomly allocated to either self-management education as provided by the existing programme (usual care)or to self-management education plus a mobile phone self-management support intervention. Participants in both arms complete assessments at baseline, one year and two years after inclusion.

### Ethical approval

Medical ethical approval for this study was obtained from the Institutional Review Board of the Institute of Tropical Medicine Antwerp (11245776), the Medical Ethics Committee of the *Universitair Ziekenhuis Antwerpen* (B300201111924), the National Ethics Committee for Health Research in Cambodia (207 NECHR), the University of Kinshasa in the Democratic Republic of Congo (ESP/CE/050/11) and the Veterans Memorial Medical Centre in the Philippines (VMMC-2011-012).

### Study context and population

The target population consists of persons with diabetes who are presently participating in existing DSME programmes in the DRC, Cambodia and the Philippines. The overall prevalence of diabetes in DRC is estimated between 3.2%, in Cambodia at 2.9% and in the Philippines at 10.0% (IDF [20,21]). These existing DSME programmes have been developed in reaction to their surrounding health system and its wider social, cultural and economic context [22,23]. To some extent, the three DSME programmes could be explained as exemplary for their context.

In DRC, the study context is an established network of 60 primary care first line centres for diabetes care in Kinshasa, in which specialised nurses, referred to as educators, act to implement the DSME programme. Five centres have been purposively selected to recruit study participants. Similar to other countries in Sub-Saharan Africa, professional care for people with diabetes is usually provided at health services [24,25]. The DSME programme in the Philippines is provided by a number of specially trained ‘Barangay’ or community health workers and/or nursing aides/midwives as educators in Quezon City (Metro Manila), in the City of Batac (Ilocos Norte Province) and in the municipality of Pagudpud (Ilocos Norte Province). Health systems in Asian countries have a long-standing tradition of such community health workers in the delivery of primary care. The DSME programme in Cambodia is facilitated through community-based peer educator networks, which began in 2005 as a relatively new development. Peer educators are responsible for the DSME programme for patients who live in their area, supported by a headquarter office in Phnom Penh. Nine peer educators have been purposively selected from one urban network and 6 rural networks in 2 provinces (Kampong Speu province, Takeo province).

**Figure 1:**
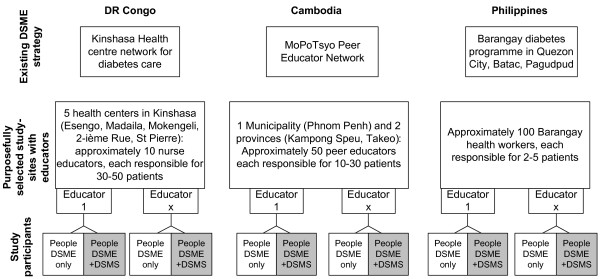
Context, study sites and block randomisation of the TEXTFORDSM study.

Because of differences in the existing DSME programmes in each country, the numbers of diabetics cared for by one educator are different in DR Congo, Cambodia and the Philippines. The purposive selection of participating centres in each country is based upon comparable patient size, quality of DSME programme, willingness of DSME programme staff to be part of a research project and convenience factors such as travel distance. Figure 1 shows how the design is implemented in each country.

### Sample size calculation

The primary outcome measure on which the sample size calculation is based is the difference in the proportion of diabetics with a well controlled HbA1C (defined as HbA1C <53 mmol/mol - comparable with 7.0% [26]) between the intervention and the usual care group. We consider a difference in HbA1C of 15% between the intervention and the usual care group after 24 months as relevant and we assume that at the start of the intervention, 60% of the participants has a well-controlled HbA1c. Unlike most interventions reporting results in terms of decrease in mean HbA1C levels [27], we chose our outcome parameter at group level (% of people with well controlled HbA1C), because this is more relevant from a program perspective. There is very little information about the current levels of HbA1C control in cohorts of people with diabetes either in Africa or in Asia [28,29]. We based our assumption on a study from China, in which 60% of people with diabetes in a hospital cohort had well controlled HbA1C levels [30]. To reach a 2-sided significance level of 5% with 80% power, and adjusting for 10% drop-out rate, the trial needs 240 participants in each arm (intervention and control group), meaning a total of 480 study participants – in each country.

### Enrolment of participants

Eligible are people above 18 who have been diagnosed with diabetes based on the WHO/IDF guidelines who are listed in a centre participating in the study and have received at least one session with an educator in the DSME programme in the last year. There are no additional exclusion criteria. All people in the participating centres, patients and staff, will be informed about the study through announcements, written materials and meetings and they will be invited to participate. In DRC and Cambodia , enrolment is carried out in a serial way, during consecutive days planned for each participating centre. Because of the smaller size of the educator’s groups, enrolment in the Philippines will be done in a droplet way. The randomisation system uses a 4X4 block randomised block design with the participant as unit of randomisation. Study code numbers and randomisation envelopes are prepared prior to enrolment. Persons willing to join will be subject to an informed consent procedure. After signing the informed consent form, they will be assigned a code number and be allocated to either trial arm. This procedure will be led by the study team, independently from the staff in the participating centres. Randomisation is blinded, but the nature of the intervention leads participants to know to which arm they belong. Educators and other staff of the participating centres may get to know this information through the participants as well.

### Usual care: the diabetes self management education programme

The project introduces a DSMS intervention in addition to an existing DSME programme. Before the DSMS intervention starts, all country teams will have optimised their existing DSME-programme up to the level of a ‘minimum package’. This comprises a coherent story explaining diabetes for patients and key messages about nine specific dimensions of disease management: 1) explanation of diabetes; 2) healthy eating; 3) physical activity; 4) monitoring; 5) medications; 6) foot care; 7) tobacco and alcohol control; 8) patient-held records; and 9) problem solving by and empowerment of patients [31,32]. The minimum package has been defined through a consultation process with people from the existing DSME projects and with diabetologists providing expert advice. The minimum package is locally validated and fine-tuned to the local context by each country team, in collaboration with local project staff, health care providers and patients. There will be a written curriculum of the DSME programme in each country.

### Intervention

The content and process of DSMS is based upon the minimum DSME package and on elements of behaviour theory, aiming to influence the modifying factors of behaviour. DSMS messages can, for instance, increase knowledge about certain behaviours and their effects, they can create normative beliefs and social pressure and they can provide emotional support and increase the perception of control by people with diabetes. Messages for DSMS will follow the nine dimensions of DSME, but will be locally developed and validated through consultations with DSME staff and people with diabetes. The local validation pertains to the local meaning of health behaviours (for instance what is a healthy diet) but also to locally relevant modifying factors of behaviour (constraints for doing physical exercise). The participants allocated to the intervention group will receive a regular mobile phone. They will receive standardised and individualised Project-Initiated Communication (ProjIC) through Short Messaging Services (SMS), which implies the maximum length of messages being 160 characters. The software FrontLine is used to send SMS in an convenient way. Furthermore, people are encouraged to use the phone to contact other people including fellow patients, educators and providers to ask for advice or provide information when needed, for instance when they cannot come to an appointment. Depending on the arrangements with the national telephone providers, participants will be provided with a budget for calls and messages. This is termed Patient-Initiated Communication (PatIC).

To control for the potential effect of providing participants with a regular mobile phone, the participants allocated to the usual care group will also receive a mobile phone but will not receive ProjIC. They will however continue to receive assistance from their educators according to the DSME programme.

The educators are not involved in the initiation of DSMS-related communication. However, all educators will be provided with a regular mobile phone so that they can be reached by patients. They will receive a modest monthly allowance to compensate them for the additional work and costs that they will have because of the intervention: receiving more calls/SMS from patients; taking necessary action upon these; and being involved in the data collection for physical examination and blood sampling. They will continue to provide DSME to their patients irrespective of their allocation to one of the trial arms.

The study project manager in each country is the central person in providing ProjIC to the people in the DSME+DSMS group. He/she will be provided with a smart phone with instructions and training on how to use it. He/she will enter patient-related data into a database that is developed and managed at national level and send out ProjIC to patients that belong to the DSME+DSMS group in the different participating centres/field sites. He will support and give feedback to educators when necessary and coordinate research data collection at site level.

### Data collection

From each participant, we will collect data linked to each of the research questions and objectives. The variables are listed in Table 1 including the instruments used and the ways to collect these data. These variables will be collected for all participants at baseline, one year and two years after inclusion by trained research staff who will interview participants guided by written questionnaires, and who will perform physical examination and collect blood samples.

**Table 1:** List of variables, measuring instruments and data sources

	**Variable**	**Instrument**	**Data collection method**
**Health outcome indicators**			
	HbA1C	DCA Vantage (Siemens)	Blood samples taken by trained field researchers, analysed by laboratory staff at location. Results on a patient record form
	Blood pressure	Electronic sphygmomanometer from certified manufacturer, study Standard Operating Procedure (SOP)	By trained field researchers, on patient record form
	BMI	Height and weight measured by research staff, study SOP	By trained field researchers, on patient record form
	Waist/Hip ratio	Conventional measuring tape according to study-SOP	By trained field researchers, on patient record form
	Diabetic foot lesions (nr)	Number of active wounds on both feet, detected through systematic examination by trained research staff	By trained field researchers, on patient record form
**Access to care indicators**			
	Attendance of DSME and medical care	Self-report over 12-month period: number of educator-contacts (check DSME patient records), visits to medical doctor, kidney-function test	Interviews with paper questionnaire, by researchers (Q28, 30, 31)
	Total medical expenditure	Self-report	Interviews with paper questionnaire, by researchers (Q32 a+b)
	Total time for h care visits	Self-report	Interviews with paper questionnaire, by researchers ( Q32c+d)
**Quality of support indicators**			
	Perceived Quality of Care DSME	PACIC score (Glasgow et al. [33])	Interviews with paper questionnaire, by researchers (Q33a-t)
	DSME needs and services	6-elements questionnaire, inspired by Diabetes Care Profile [34]	Interviews with paper questionnaire, by researchers (Q34+35)
	Enablement through DSME	Patient enablement score [35]	Interviews with paper questionnaire, by researchers (Q36)
**Empowerment indicators**			
	Level of knowledge on diabetes (care)	Diabetes Knowledge Score, adapted from Brief Diabetes Knowledge Test & the Diabetes Knowledge Questionnaire ([36,37])	Interviews with paper questionnaire, by researchers (Q13)
	Attitude towards diabetes	Attitude scale Diabetes Care Profile [34]	Interviews with paper questionnaire, by researchers (Q14)
	Feeling of control	Control scale from Diabetes Care Profile [34]	Interviews with paper questionnaire, by researchers (Q15a-d)
**Self-management behaviour**			
	Adherence to glucose monitoring & control	Self-report of last time self-monitoring, professional monitoring, medication taking	Interviews with paper questionnaire, by researchers (Q16-25)
	Adherence to exercise & diet	Self-report	Interviews with paper questionnaire, by researchers (Q38/37, 41)
**Process indicators**			
	Number of SMS sent (ProjIC) to participant	Database that is used to send out ProjIC	Assessment by the project manager
	Number and type (call or SMS) of PatIC to educators	Telephone provider records	Assessment by the project manager
	intervention cost at DSMS programme level	Extraction from data from project documents	Collected by country research team
**Context characteristics**			
Diabetes prevalence, phone penetration, literacy rate, DSME programme characteristics	extraction from data from project documents, country-related publications and other secondary data	Collected by country research team	
	Knowledge and professional habits of diabetes care providers	Provider Profile, Diabetes Management Practices and Brief Diabetes Knowledge & Practice Test (adapted from ([38])	Written self-administered questionnaire to health providers

The patient questionnaire (Additional file 1) includes several scales that measure dimensions of chronic care: the ‘needs & services-scale’, ‘attitude scale’ and ‘control scale’ which are all subscales from the Diabetes Care Profile (DCP) [34]; the ‘patient enablement score’ [35]; and the Patient Assessment of Chronic Illness Care (‘PACIC Score’) [39]. These scales have been validated in heterogeneous populations in clinical and community settings in western countries [34,33]. The ‘patient enablement score’ has been validated in a low income country [40]. The questionnaire was developed for a study involving persons with diabetes in the Philippines. Results of that study are yet to be published. The questionnaires will be translated into local languages and pretested in all countries. We will also collect the following personal and diabetes (care) related variables: age, sex, education, diabetes history (year of diagnosis, hospital admissions, treatment, hypertension) and physical access to care (distance to care providers). For the process analysis, we will assess the number of SMS sent to each participant in the intervention group and the number of phone calls / SMS that participants of both groups made to the educator.

For each country, we collect a number of contextual characteristics from primary and secondary sources. These are: prevalence of diabetes, mobile phone penetration, literacy rate, details about the existing DSME programme (number of patients per educator, components of DSME strategies, frequency of contact between patients and educator, etc.), presence of patient organisations and knowledge and professional habits of diabetes care providers (through self-administered questionnaire sent to diabetes care providers in the environment).

Data will be electronically entered and cleaned through Epi-Info, using a double-entry procedure.

### Data analyses

Analyses of quantitative data including assessment of differences in changes in primary and secondary outcomes between the intervention and usual care group will be done making use of Stata version 11. A probability of <0.05 will be considered statistically significant for all tests. Continuous variables will be tested for normality and non-normal distributions will be categorised or transformed. Descriptive analyses will be performed for all variables and unadjusted comparisons between study groups will be made using T-tests (for continuous variables) or Chi-square tests (for discrete variables). Outcome indicators will be plotted over time. All data will be analysed for confounding and interaction in multivariate regression analyses taking potential clustering effects into account.

The primary endpoint of the study is the proportion of participants with a well controlled HbA1C (< 53 mmol/mol) at the last available time-point after the start of the intervention, normally 24 months after inclusion. In case this measurement is not available due to patients transferring to another health service, migration, missed measurements or unavailable samples, an earlier time point (from month 21 onwards) can be carried forward. Patients who died, stopped the study due to diabetes-related morbidity, were lost to follow up (i.e., dropped out of the study for unknown reasons), or refused further continuation in the programme or the study will be counted as failures and will not be included in the analysis. The proportion of patients with well-controlled HbA1C will be compared between intervention and control groups using a logistic regression model with fixed effects for intervention (yes/no), HbA1C control status at baseline (just before initiation of the intervention), and educators. Intervention effects will be tested in this model with a two-sided significance level of 5%. Additionally, we propose to analyse the repeated outcomes (at the different time points), using longitudinal (mixed effects or GEE) logistic regression models to determine temporal trends in the data.

The main secondary variables of interest are: difference in change of mean blood pressure, mean BMI and mean waist/hip ratio. Secondary variables will be assessed using similar logistic regression or ANOVA/regression (for continuous data) models. Outcome indicators will be plotted over time. Repeated measure models will be used to analyse longitudinal trends over time or to correct for missing data and hierarchical models will be used to assess the influence of educator characteristics on the effects.

Recruitment will be evaluated by assessing the number of individuals who are: part of the target population informed about the study and requested to participate; meeting the inclusion criteria; having signed the informed consent; and having been enrolled in one of the study arms. Loss-to-follow up will be measured by subtracting the number of individuals in each arm of the study returning for evaluation at determined moments of evaluation from the total number of participants at inclusion.

### Multi-country analysis

After the analysis of the effectiveness of the intervention in each country, we will perform a multi-country analysis. We will analyse if any statistically significant differences in the primary and secondary end-points can be detected between the three countries. We will use a realistic approach to look for factors explaining any identified differences between countries, explaining how the actual intervention led to the observed results and why the results differ (or not) between countries [41-43]. Project documents, project reports from the field sites and observation reports will be collected. In-depth interviews will be carried out with project managers and diabetes educators and focus group discussions with patients from both groups will be carried out. Triangulation of data will be done by comparing the results from these sources. The interview recordings will be transcribed verbatim and entered in N-Vivo. Qualitative data analysis will be carried out using the programme theory as the analytical framework [44,45].

### Cost-effectiveness analysis

In addition, we will conduct an incremental cost-effectiveness analysis (CEA) comparing the DSMS strategy in addition to the existing DSME strategy with the DSME strategy only. The evaluation will take the societal viewpoint taking into account both patient and programme costs. Costs incurred by patients consist of direct medical (e.g. cost for consultation, medication and the cost for urgent care and hospitalisation), direct non-medical (cost for using mobile phone for PatIC, travel cost, food cost, cost related to exercise) and indirect costs. Indirect costs include the value of time spent by participants visiting educators and other health care providers and the loss of productivity due to illness. Programme costs include the cost for development and management of the intervention and the cost for telecommunication. Information about patient direct and indirect costs will be collected by a trained field researcher with a patient questionnaire (see section on data collection). The value of time losses will be estimated from secondary sources taking the GDP of the three countries into account. The information on programme costs will be collected prospectively by keeping a detailed account of all expenditures at the programme level. Programme effectiveness will be expressed in terms of the percentage increase of people with diabetes with a controlled HbA1C level [46,47].

## Discussion

The central hypothesis that will be tested in this study is that a mobile phone DSMS intervention on top of an existing DSME programme will improve clinical outcomes for people with diabetes, measured by their HbA1C level. The wide range of secondary variables will yield information on intermediary outcomes and on other outcomes which are also very relevant for the organisation of support.

Although the intervention itself is relatively simple, its effects are realised through complex processes, like the behaviour (change) of people with diabetes. The effect of the intervention will partly depend on factors related to the process itself (for instance, the level of interaction and personalisation of messages), but also on the profile of people with diabetes (for instance their attitude towards their diabetes and their familiarity with mobile phones) and on the context [48,49].

The multi-country analysis will aim to understand any differences in the effects of the intervention and the role of the context, for instance the design of the existing DSME programme, knowledge and professional habits of diabetes care providers.

The major limitation of our design with individual randomisation at the level of the patient is that within each participating centre, there will be patients in the DSME-only group and in the DSME+DSMS group. Patients can be in contact with each other and this might cause some contamination between DSME-only and DSME+DSMS patients, resulting in a sub-estimation of effect. The individual targeting of the messages and the central management of the DSMS intervention without interference of the educators should minimise this contamination. Another methodological weakness is the lack of local validation of the scales within the questionnaire, which fell beyond the scope of our study. Pretesting and local fine-tuning will partly address this issue. Since we will use the same questionnaire 3 times along the course of the study, we expect that familiarity with the instruments will grow over time.

The growing numbers of patients with chronic life-long conditions, such as diabetes and hypertension, puts an immense burden on health systems and populations [50]. Scarcity of resources and the lack of quality and continuity of health care result to high expenditure and very poor health outcomes. The intervention tested addresses the problem that the greater part of diabetes management takes place without external support and that many challenges, unforeseen problems and questions most of the time occur at the “in-between moments” of the scheduled contacts with the support system, like health care providers and educators. The project exploits the availability of widely accessible equipment and communication technology to narrow the gap between the support system and people with diabetes. In this way, it addresses the need of people with diabetes to combine their life-long condition with their other needs and roles in life and to contribute to their empowerment [51,52]. It will be interesting to evaluate the impact of this complementary DSMS strategy on the workload of the educators and health provides, an important issue for further scaling up in general services (WHO [53]). The study addresses gaps in knowledge and experience on utilization of mobile phone technology to support people with diabetes in developing countries. Results will provide information to decision-makers regarding conditions of implementation in developing countries and possible expected results.

## Abbreviations

DSMS: Diabetes self-management support; DSME: Diabetes self management education

## Competing interests

Kristien Van Acker has a function in the Diabetes Foot Programme of IDF.

None of the authors has any financial competing interest.

## Authors’ contributions

JVO is coordinating investigator of the study and drafted the first version of this text. JVO, GMK, MVP, JCK, HH, CD, BV, GK have been all involved in the design of the project, the writing of the protocol and its implementation. GMK designed the patient questionnaire. FS and KVA have provided specialist advice about diabetes and methodology. All authors have contributed to the manuscript by providing content-related feedback and improvements on draft versions. All authors read and approved the final manuscript.

## Pre-publication history

The pre-publication history for this paper can be accessed here:

http://www.biomedcentral.com/1471-2458/13/423/prepub

## Supplementary Material

Additional file 1 **Webannex 1.** Patient Questionnaire. Click here for file
