# Supplementary material for: The effectiveness of text messages support for diabetes self-management: protocol of the TEXT4DSM study in the democratic Republic of Congo, Cambodia and the Philippines
Source: BMC Public Health. 2013 May 1;13:423. doi: 10.1186/1471-2458-13-423 (PMC3658970; doi:10.1186/1471-2458-13-423)
Supplement: Additional file 1 — Webannex 1. Patient Questionnaire. [file 1471-2458-13-423-S1.doc]

| Diabetes Support Study |
| --- |
| *Patient Questionnaire*  *Version 2.3 FINAL & tested*  *Similar to back-page Epi-info* |
|  |

| **Study Code Number** |  | |
| --- | --- | --- |
| **Main health provider** |  | |
| **Diabetes Educator** |  | |
| **Interviewer/Examiner's Name** |  | |
| **Evaluation round** | **Round** | **1** |
|  |  | **2** |
|  |  | **3** |
| **Date (dd/mm/yyyy)** | _____/_______/_______ | |

*(This table is not to be filled in by interviewer)*

|  |  |  |  | (DD) | (MM) | (YYYY) |
| --- | --- | --- | --- | --- | --- | --- |
| *Data Entry Clerk 1 - Name* |  |  | *Date* |  |  |  |
| *Data Entry Clerk 2 - Name* |  |  | *Date* |  |  |  |
| *Quality Control Officer (if applicable) - Name* |  |  | *Date* |  |  |  |

| **GENERAL DATA (only to fill in at baseline evaluation)** | | | | |
| --- | --- | --- | --- | --- |
|  | Birth year | _________(year) | | Write |
|  | Sex | Male | 1 | Encircle the answer |
|  |  | Female | 2 |  |
|  | Level of education | Illiterate (can neither read nor write) | 1 | Encircle the answer |
|  |  | Primary | 2 |  |
|  |  | Secondary | 3 |  |
|  |  | University | 4 |  |
|  |  | Not known | 9 |  |
|  | How long do you travel to your educator? | _____(hours) | Write | |
|  | How long do you travel to your doctor? | _____(hours) | Write | |
|  | What year were you first told you had diabetes? | ____(year) | Write | |

| **DIABETIC CONDITION** | | | | |
| --- | --- | --- | --- | --- |
|  | Have you ever been admitted to a hospital/clinic due to diabetes or hypertension? | Yes | 1 | Encircle the answer |
|  |  | No | 2 |  |
|  | What was the reason necessitating admission? | Hypoglycaemia | 1 | Multiple answers possible |
|  |  | Infection | 2 |  |
|  |  | Stroke | 3 |  |
|  |  | Heart disease | 4 |  |
|  |  | Kidney disease | 5 |  |
|  |  | Eye problem | 6 |  |
|  |  | Hypertension | 7 |  |
|  |  | Others. Specify_________________ | 8 |  |
|  |  | Not relevant | 9 |  |
|  | What is the **treatment** that is **prescribed** to you for the management of your diabetes? | Diet and/or exercise. | 1 | Multiple answers possible |
|  |  | Oral medication:  If yes: _________________(name)  _________________(name) | 2 |  |
|  |  | Insulin.  If yes, how many times per day?  __________(times/day) | 3 |  |
|  | What is the **treatment** that **you take now** for the management of your diabetes | Nothing | 1 | Multiple answers possible |
|  |  | Diet and/or exercise. | 2 |  |
|  |  | Oral medication:  If yes: _________________(name)  _________________(name) | 3 |  |
|  |  | Insulin.  If yes, how many times per day?  __________(times/day) | 4 |  |
|  | Have you been prescribed medication for **hypertension**? | Yes, _________________(name) | 1 | Encircle the answer |
|  |  | No | 2 |  |
|  | Do you take it now? | Yes | 1 | Encircle the answer |
|  |  | No | 2 |  |

# ENABLEMENT & SELF MANAGEMENT

| **KNOWLEDGE** | | | | |
| --- | --- | --- | --- | --- |
|  | **Questions** | **Yes** | **No** | **I don’t know** |
|  | The usual cause of diabetes is lack of effective insulin in the body |  |  |  |
|  | In untreated diabetes, the amount of sugar in the blood usually increases |  |  |  |
|  | If I am a diabetic, my children have a higher chance of being diabetic |  |  |  |
|  | Diabetes can be cured |  |  |  |
|  | A sugar level of 210 in the blood before eating is too high  (Philippines: A sugar level of 210 in the blood in too high) |  |  |  |
|  | Exercise and eating influence the need of insulin |  |  |  |
|  | There are 2 main types of diabetes |  |  |  |
|  | Healing of cuts and abrasions in diabetics is the same as in people without diabetes |  |  |  |
|  | Diabetics should take extra care when cutting their toenails  (Cambodia: other question checking knowledge about footcare) |  |  |  |
|  | The way I prepare my food, for instance which additives and oil I put, is as important as the foods I eat |  |  |  |
|  | Diabetes can damage my kidneys |  |  |  |
|  | Diabetes can cause loss of feeling in my hands, fingers, and feet |  |  |  |
|  | Shaking and sweating are signs of high blood sugar |  |  |  |
|  | Frequent urination and thirst are signs of low blood sugar |  |  |  |
|  | Healthy food for diabetics can be found at the local market |  |  |  |
|  | A diabetic should also limit his salt and intake of bad fats |  |  |  |
|  | Fruit juice without any added sugar raises blood sugar |  |  |  |
|  | Diet softdrinks (Phil/DRC) / Hot tea (Cam) can be used to treat low blood sugar |  |  |  |
|  | A diabetic is more at risk to develop heart disease or suffer from a stroke |  |  |  |
|  | Infection/Fever is likely to cause the blood sugar to decrease |  |  |  |

|  | **ATTITUDE TOWARDS DIABETES** | |
| --- | --- | --- |
|  |  | Encoding |
|  | I am afraid of my diabetes |  |
|  | I find it hard to believe that I really have diabetes |  |
|  | I feel unhappy and depressed because of my diabetes |  |
|  | I feel satisfied with my life |  |
|  | I feel I am not as good as others because of my diabetes |  |
|  | I can do most things that I want to do / I can lead a normal life. |  |
|  | I find it hard to do all the things I have to do for my diabetes |  |
|  | Diabetes does not affect my life at all |  |
|  | I am pretty well off, all things considered |  |
|  | Things are emotionally going very well for me right now |  |
|  | I feel dissatisfied with my life because of my diabetes |  |

| **FEELING OF CONTROL** | |  |
| --- | --- | --- |
|  | I’m able to ….. | Encoding |
|  | Keep my blood sugar in good control |  |
|  | Keep my weight under control |  |
|  | Do the things I need to do for my diabetes (diet, exercise, take medicine) |  |
|  | Handle my feelings (fear, worry, anger) about my diabetes |  |

| **GLUCOSE MONITORING** | | | | |
| --- | --- | --- | --- | --- |
| **GLUCOSE MONITORING** | | | | |
|  | Do you check your glucose levels yourself? | Yes | 1 | Encircle the answer |
|  |  | No | 2 |  |
|  | How do you check your glucose levels | In urine | 1 | Multiple answers possible |
|  |  | In blood | 2 |  |
|  |  | Not relevant | 9 |  |
|  | How often? |  | | Write (if not relevant, write 0) |
|  | When was the last time that you **yourself** checked your glucose? | ____/_____/_______(date, dd/mm/yyyy) | | Write (if not done, write 00/00/000) |
|  | When was the last time that your glucose was checked by an **educator/other professional**? | ____/_____/_______(date, dd/mm/yyyy) | | Write |

| **GLUCOSE CONTROL** | | | | |
| --- | --- | --- | --- | --- |
|  | How do you control your blood sugar level? | Excercise and/or diet | 1 | Multiple answers possible |
|  |  | I take oral medication | 2 |  |
|  |  | I use insulin | 3 |  |
|  |  | Others: Specify________________________ | 4 |  |
|  | Do you normally take any medication for your diabetes? | Yes | 1 | Encircle answer |
|  |  | No | 2 |  |
|  | Why do you not take medication for diabetes condition? | I don’t need to (control blood sugar through diet and exercise) | 1 | Multiple answers possible |
|  |  | I don’t want to myself | 2 |  |
|  |  | It is too expensive | 3 |  |
|  |  | Not available | 4 |  |
|  |  | Others: Specify_________________________ | 5 |  |
|  |  | Not relevant | 9 |  |
|  | How often in a day are you required to take your medicine? | Once a day | 1 | Encircle answer |
|  |  | Two times a day | 2 |  |
|  |  | Three or more times a day | 3 |  |
|  |  | Not relevant | 9 |  |
|  | Do you take your medicines as they are prescribed? | Yes |  |  |
|  |  | No |  |  |
|  | Reasons for not taking medications according to the prescription. | I forget to take medicine | 1 | Multiple answers possible |
|  |  | I am unable to buy all prescribed medicine | 2 |  |
|  |  | I could not eat and therefore did not take the medicine | 3 |  |
|  |  | I experience side effects | 4 |  |
|  |  | Others, specify___________________ | 5 |  |
|  |  | Not relevant | 9 |  |

# DIABETES SELF-MANAGEMENT EDUCATION PROGRAMME

| **ATTENDANCE** | | | | |
| --- | --- | --- | --- | --- |
|  | How often are you supposed to meet with your educator?  (DRC: nurse  Cam: peer educator  Phil: Barangay) | Every week | 1 | Encircle the answer |
|  |  | Every 2 weeks | 2 |  |
|  |  | Every month | 3 |  |
|  |  | Every 2 months | 4 |  |
|  |  | Every 3 months | 5 |  |
|  |  | Every 4 months | 6 |  |
|  |  | Every 6 months | 7 |  |
|  | How many times have you met with your educator in the last year? | _______(times) | | Write |
|  | If you did not see him/her, what was the reason for this | Too far | 1 | Encircle the answer |
|  |  | Too expensive | 2 |  |
|  |  | No time / Inconvenient time | 3 |  |
|  |  | Quality of care not sufficient | 4 |  |
|  |  | No need to go | 5 |  |
|  |  | Others, Specify___________________________________________________ | 6 |  |
|  |  | Not relevant | 9 |  |
|  | How often have you seen a medical doctor for your diabetic condition in the last 6 months? | _______(times) | | Write |
|  | Did you have your kidney function tested in the last year? | Yes | 1 | Encircle the answer |
|  |  | No | 2 |  |
|  |  | I don’t know | 3 |  |

| **HEALTH CARE EXPENDITURE IN THE LAST MONTH** | | | |
| --- | --- | --- | --- |
|  | **In the last month, how much did you spend on ….** | | |
|  | Consultation, medication, urgent care and hospitalization? | __________(local currency) | Write |
|  | On other issues which are connected with your diabetic condition, such as telephone use, travel, special food, exercise, etc | __________(local currency) | Write |
|  | How much time did you spend to visit the educator, including traveltime?  (DRC = nurse; Cam = peer educator; Phil = Barangay) | _________(hours) | Write |
|  | How much time did you spend to visit health provider(s), including traveltime? | _________(hours) | Write |

| **PERCEIVED QUALITY OF CARE** | |  |
| --- | --- | --- |
| **Think of the meetings that you have had with your educator and the other meetings that are part of the diabetes programme of which you are part and answer the following 20 questions, remembering those meetings.** | |  |
|  | **“In the time that I have been in the diabetes programme and during meetings with my educator, I was ….”** | **Encoding** |
|  | Asked for my ideas when we made a plan for treatment |  |
|  | Given choices about treatment to think about. |  |
|  | Asked to talk about my problems with my medicines or their effect. |  |
|  | Given written material about how I should improve my health. |  |
|  | Of the opinion that my care was well-organized. |  |
|  | Shown how what I did to take care of my illness influenced my condition......... |  |
|  | Asked to talk about my goals in caring for my illness. |  |
|  | Helped to set specific goals to improve my eating or exercise habits. |  |
|  | Given a copy of the plan for treatment. |  |
|  | Encouraged to go to a specific group or class to help me cope with my illness. |  |
|  | Asked questions about my health habits. |  |
|  | My values and traditions were respected when they recommended treatments to me. |  |
|  | Helped me to make a treatment plan that I could do in my daily life. |  |
|  | Helped me to plan ahead so I could take care of my illness even in difficult times or stressful situations. |  |
|  | Asked how my chronic illness affects my life / (Camb version: “My diabetes educator knows how I feel about my diabetes and how it affects my life.”) |  |
|  | Contacted after my visit to see how things are going |  |
|  | Encouraged to attend programs in the community that could help me. |  |
|  | Referred to another health care giver. |  |
|  | Given specific instructions on how I can take care of myself better, for instance conducting self-foot exam or testing glucose in my urine myself. |  |
|  | Told how my visit with other types of doctors, like the eye doctor, would help my treatment. |  |

| **DIABETES SELF MANAGEMENT EDUCATION** | |  |
| --- | --- | --- |
| **34.** | **I want to have advice in…** | Encoding |
| 1. 3 | Following my meal plan / the food pyramid. |  |
|  | Taking my medicine |  |
|  | Taking care of my feet |  |
|  | Getting enough physical activity |  |
|  | Testing my sugar |  |
|  | Handling my feelings about diabetes |  |
|  | **My diabetes educator gives me advice in…** | Encoding |
|  | Following my meal plan |  |
|  | Taking my medicine |  |
|  | Taking care of my feet |  |
|  | Getting enough physical activity |  |
|  | Testing my sugar |  |
|  | Handling my feelings about diabetes |  |

| **ENABLEMENT THROUGH EDUCATOR CONTACTS**  **(Adapted Howie score)** | |  |
| --- | --- | --- |
| **“As a result of your visits to the educator, you find you are ….”** | |  |
|  |  | **Encoding** |
|  | Able to cope with life |  |
|  | Able to understand your illness |  |
|  | Able to cope with your illness |  |
|  | Able to keep yourself healthy |  |
|  | Confident about your health |  |
|  | Able to help yourself |  |

| **HEALTHY LIFESTYLE** | | | | |
| --- | --- | --- | --- | --- |
|  | How much do you walk each day? | 20 minutes or less less than 20 minutes | 1 | Encircle the answer |
|  |  | 20 minutes – less than an hour | 2 |  |
|  |  | 1-2 hours | 3 |  |
|  |  | More than 2 hours | 4 |  |
|  | How often do you engage in (other) physical exercise? | Everyday | 1 | Encircle the answer |
|  |  | Several times a week | 2 |  |
|  |  | Several times a month | 3 |  |
|  |  | Less than once a month | 4 |  |
|  | What type of physical exercise do you engage in? | Aerobic exercise (e.g.,brisk walking, jogging, swimming, cycling) | 1 | Multiple answers possible |
|  |  | Weightlifting | 2 |  |
|  |  | Others,  Specify___________________________________________________ | 3 |  |
|  |  | Not relevant | 9 |  |
|  | Reason(s) for not engaging in regular physical exercise | Lack of time | 1 | Multiple answers possible |
|  |  | Lack of money | 2 |  |
|  |  | Health condition prevents me from doing physical exercise | 3 |  |
|  |  | Others, Specify___________________________________________________ | 4 |  |
|  |  | Not relevant | 9 |  |
|  | Do you follow a diabetic diet? | No | 1 |  |
|  |  | Sometimes | 2 |  |
|  |  | Yes/Most of the time | 3 |  |
|  |  | Don’t know what is diabetic diet | 4 |  |
|  | Reasons why I do not always follow a diet | I don’t want to | 1 | Multiple answers possible |
|  |  | It is difficult to prepare | 2 |  |
|  |  | Lack of self-control | 3 |  |
|  |  | Poor support from family members | 4 |  |
|  |  | Others, Specify___________________________________________________ | 5 |  |
|  |  | Not relevant | 9 |  |

| **INFORMATION** | | | | |
| --- | --- | --- | --- | --- |
|  | Where do you get information regarding diabetes and control of blood sugar? | Media (Newspaper/TV/Radio/internet) | 1 | Multiple answers possible |
|  |  | Other people with diabetes | 2 |  |
|  |  | My educator (nurse/peer/Barangay) | 3 |  |
|  |  | Healthcare provider | 4 |  |
|  |  | Written brochures/booklets | 5 |  |
|  |  | Others, Specify____________________________________________ | 6 |  |
|  |  | Not relevant | 9 |  |
|  | When you have questions relating to your diabetes condition, whom do you usually ask? | Search on my own for answers | 1 | Multiple answers possible |
|  |  | Ask neighbours/friends for advice | 2 |  |
|  |  | Ask other diabetes patients for advice | 3 |  |
|  |  | Ask educator (nurse/peer/Barangay) | 4 |  |
|  |  | Ask doctor/health provider | 5 |  |
|  |  | Others, Specify____________________________________________ | 6 |  |
|  |  | Not relevant | 9 |  |
|  | Are you part of an organised group of patients with diabetes? | Yes | 1 | Encircle the answer |
|  |  | No | 2 |  |
|  | How often do you attend activities of this group? | Several times per week | 1 | Encircle the answer |
|  |  | Several times per month | 2 |  |
|  |  | Less than once a month | 3 |  |
|  |  | Less than once a year | 4 |  |
|  | What are the activities that the group organises? | Social get together | 1 | Multiple answers allowed |
|  |  | Education and advice among each other | 2 |  |
|  |  | Look for somebody who has been absent | 3 |  |
|  |  | Others, Specify____________________________________________ | 4 |  |
|  |  | Not relevant | 9 |  |

# RESOURCES FOR INFORMATION

| **MOBILE PHONE** | | | | |
| --- | --- | --- | --- | --- |
|  | Do you **have** a mobile phone? | Yes | 1 | Encircle the answer |
|  |  | No | 2 |  |
|  | What sort of phone is it? | Regular cellular phone | 1 | Multiple answers possible |
|  |  | Smartphone/Iphone | 2 |  |
|  | How do you use it **in general** (not only related to your diabetes condition)? | To make telephone calls | 1 | Multiple answers possible |
|  |  | To receive telephone calls | 2 |  |
|  |  | To send and/or receive short message services (SMS) | 3 |  |
|  |  | Others,  Specify____________________________________________ | 4 |  |
|  |  | Not relevant | 9 |  |
|  | How often do you use the phone in general? | Several times per day | 1 | Encircle the answer |
|  |  | Several times per week | 2 |  |
|  |  | Less than once a week | 3 |  |
|  |  | Not relevant | 9 |  |
|  | How often have you used your phone (phone call or SMS) **for your diabetes** during the last month? | ________(number of times) |  | Write. |
|  | To which persons did you **phone/send SMS** mentioned above? | Neighbours/friends | 1 | Multiple answers possible |
|  |  | Other diabetes patients | 2 |  |
|  |  | Educator | 3 |  |
|  |  | Doctor/health provider | 4 |  |
|  |  | Others,  Specify____________________________________________ | 5 |  |
|  |  | Not relevant | 9 |  |
|  | How often have you **received** a phonecall / SMS from your educator in the last month?  (nurse/peer/Barangay) | _________(number of times) |  | Write. |
|  | How often have you received a phonecall / SMS from the mobile phone support project in the last month? | __________(number of times) |  | Write. |
